# Strength and Water-Repelling Properties of Cement Mortar Mixed with Water Repellents

**DOI:** 10.3390/ma14185407

**Published:** 2021-09-18

**Authors:** Hyeju Kang, Sukpyo Kang, Byoungky Lee

**Affiliations:** 1 Department of Construction Engineering, Woosuk University, Jincheon 27841, Korea; leekang02@nate.com; 2 Department of Architecture, Woosuk University, Jincheon 27841, Korea; 3 COCHEMS Co., Ltd., Industrial Tools Circulating Center, 160, Daehwa-ro, Daedeok-gu, Daejeon 34368, Korea; fluolbk@naver.com

**Keywords:** silane, siloxane, water repellents, hydrophobic, contact angle, cement mortar, rapid-hardening cement, repair mortar

## Abstract

In this study, the compressive strength and water contact angle of mortar specimens prepared by mixing two types of water repellent with ordinary Portland cement (OPC) and rapid-hardening cement mortar were measured before and after surface abrasion. In addition, the hydration products and chemical bonding of cement mortar with the repellents were examined using X-ray diffraction (XRD), thermogravimetry-differential thermal analysis (TG-DTA), and Fourier-transform infrared spectroscopy (FT-IR) to evaluate the performance of these cement mortar mixtures as repair materials. We found that the fast-hardening cement mortar mixture containing the oligomer water repellent showed the best performance with a high compressive strength and large water contact angle. With the oligomer water repellent, the rapid-hardening cement mortar mixture showed contact angles of 131° and 126° even after a 2 mm abrasion, thereby confirming that the water repellent secured hydrophobicity through strong bonding with the entire cement mortar as well as its surface. The compressive strengths were found to be 34.5 MPa at 3 h and 54.8 MPa at 28 days, confirming that hydration occurred well despite the addition of water repellent.

## 1. Introduction

Cement concrete structures exhibit relatively lower damage compared to other construction materials owing to their high strength and durability, thereby incurring low maintenance costs during their life cycle [1]. Recently, however, cases of structural damage owing to various causes, such as freezing and thawing, alkali silica reaction, and carbonation, have increased [2]. In particular, cement materials can crack when exposed to moisture because their porous capillaries absorb water and the subsequent cyclical absorption, expansion, freezing, and thawing result in a loss of durability, strength, and stability [3,4].

In recent years, there has been growing interest from the research community in utilizing superhydrophobicity to address the problem of moisture penetration in cement concrete. Superhydrophobicity, a phenomenon observed in lotus leaves in nature, causes water droplets to roll down on the surface of cementitious materials, thereby reducing the degradation in durability due to moisture penetration [5]. Superhydrophobicity interferes with the presence of water and ice on concrete and prevents the penetration of corrosive ions and acids. It also prevents contact between the water and steel reinforcements in reinforced concrete structures [5].

Currently, most studies on superhydrophobicity in cement concrete have focused on creating a water-repellent layer on hardened cementitious materials by producing water repellents and coating them in a single layer or multiple layers with bonding layers, such as polymers, using spray, brushes, or rollers [6,7]. However, in these studies, hydrophobicity was developed only in those parts where the water-repellent layer was created, and the water repellency degree decreased over time, making the maintenance of hydrophobicity difficult under worn and peeled-off surfaces [8]. Recently, a few studies have reported the fabrication of superhydrophobic mortar by adding water repellents to structures for maintaining hydrophobicity [7,8,9]. In previous studies, cement and sand were coated with SiO_2_ before manufacturing cement mortar [5]; alternatively, silanized aggregate, silica fume [10], or stearic acid were used to prepare the mortar [8]. Thus, when the water repellent was mixed into the structure itself, it was found that even if micro cracks occurred, water still did not penetrate through the cracks [11]. In a recent study, a hydrophobic cement material was developed by adding isobutyltriethoxysilane (IBTEO) and nanosilica (NS) to cement, and the mechanical properties and hydrophobicity were reviewed [12]. However, there are limits to the practical use of these methods as they involve the complex pretreatment of materials and lead to low initial strength. In addition, they suffer from slow curing rates, which makes the application of such cement mortar unsuitable as a repair material in watertight structures or in rapid construction [13].

Therefore, in this study, the setting time, strength, and water contact angle of mortar samples fabricated by mixing two types of water repellents with ordinary Portland cement (OPC) and rapid-hardening cement mortar were measured before and after surface abrasion. In addition, the performance of the water repellents as repair materials was compared by examining the hydration products using X-ray diffraction (XRD), thermogravimetry-differential thermal analysis (TG-DTA), and Fourier-transform infrared spectroscopy (FT-IR).

## 2. Experimental Program

### 2.1. Materials

The two types of cement used here were OPC and rapid-hardening cement, whose physical and chemical properties are listed in Table 1. OPC (Sungshin Cement Co., Ltd., Seoul, Korea) has a specific surface area of 3144 cm^2^/g, density of 3.15 g/cm^3^, an ignition loss of 1.32, and chemical composition of SiO_2_ (21.7%), Al_2_O_3_ (5.7%), Fe_2_O_3_ (3.2%), CaO (63.1%), MgO (2.8%), and SO_3_ (2.2 %). In contrast, the rapid-hardening cement (Union Co., Ltd., Seoul, Korea) has a specific surface area of 5741 cm^2^/g and density of 2.91 g/cm^3^ with an initial setting time of 25 min and a final setting time of 0.67 h. Silica sand No. 5 with particle diameters of 1.2–0.8 mm and a mesh of 14–20 was used. Silane- and siloxane-based water repellents were used for mixing with mortar; their physical and chemical properties are listed in Table 2. The active ingredients of the siloxane (oligomer)-based water repellent are poly(dimethylsiloxane) and hydroxy-terminated poly(dimethylsiloxane), whereas those of the silane (monomer)-based water-repellent are n-octyltriethoxysilane and diethoxy-octyl-[oxo(trimethylsilyloxy)silyl]oxy-silane.

### 2.2. Experimental Plan

To assign hydrophobicity to the surface and body of the cementitious materials, an experimental plan was established as shown in Table 3. OPC (C) and rapid-hardening cement (R) were used as binders, and silica sand No. 5 (5) was used as the sand. The mix proportions were set to binder:sand:water:water repellent = 40:40:15:5, according to a previous study [5]. When water repellents were added to the mixture, the amount of water was adjusted to their 50% solid content. In addition, when rapid-hardening cement was used, 0.5% retarder (citric acid hydrate) was added to secure the time for work. In the rest of the text, mortar samples have been indicated using abbreviations according to the type of cement, sand number, and water repellent used. For example, C5-O refers to OPC, No. 5 silica sand, and oligomer water repellent.

### 2.3. Methods

For preparing the mixtures, the binders and sand were dry-mixed in a forced mortar mixer, and a mixture of water and water repellents was added. The mortar was fabricated in accordance with KS L ISO 679.

For measuring the compressive strength, cement mortar was poured into a 40 × 40 × 160 mm^3^ mold, hardened at a temperature of 20 ± 2 °C for 24 h, demolded after one day, and cured at a temperature of 20 ± 2 °C and relative humidity of 50% until the designated ages. The compressive strength was measured using three samples per mixture under the same conditions, and then the average strength value was calculated. Measurements were performed at 3, 7, and 28 days for the samples with OPC and at 3 h, 1, 7, and 28 days for those with the rapid-hardening cement in accordance with KS L ISO 679 using a universal testing machine (Cheonwang Precision, Hanam, Korea).

To examine the hydration products, samples less than 10 mm in size and aged for 28 days were collected and immersed in anhydrous ethyl alcohol for one day to stop the hydration. These samples were dried in an oven at 40 °C for 3 days.

The dried samples were crushed and passed through a 200-mesh sieve for XRD analysis (Rigaku, SmartLab, Tokyo, Japan), which was performed using a CuKa wavelength of 45 kV and 200 mA and at 4°/min for 2θ = 5–75°. The TG-DTA was conducted in air at temperatures of approximately 20–800 °C and a heating rate of 1 °C/min.

**Figure 1:**
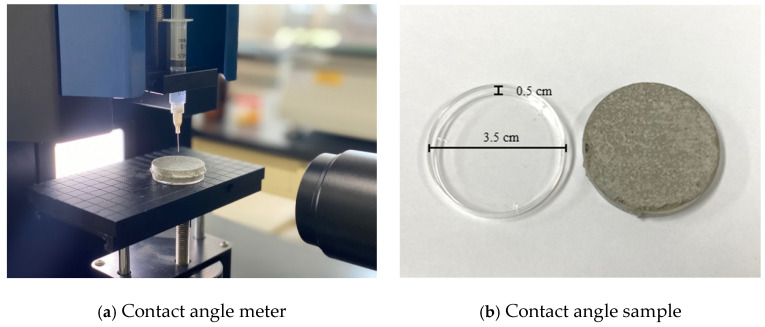
Water contact angle measurement process.

To measure the water contact angle, the mortar was poured into a Ø 3.5 cm × 0.5 cm petri dish and cured at a temperature of 20 ± 2 °C and a humidity of 50% for 28 days. After curing, the surfaces were abraded by 2 mm using a 100-grit sandpaper. The contact angles of the samples before and after sandpaper abrasion were measured using a Pico contact angle meter in accordance with the KS L 2110 wettability test method for a glass surface. Figure 1 shows the contact angle measurement and the measurement specimen.

Water absorption by capillarity (Norma UNI 10859) was investigated for three cubic samples (5 × 5 × 5 cm^3^) after curing the fabricated mortar for 28 days. The samples were immersed in water at a depth of 2–10 mm, and their masses were measured 24 h before and after immersion. The samples absorbed moisture owing to the capillary phenomenon [14,15].

Then, FT-IR spectra (Thermo, Nicolet 6700, Mundelein, IL, USA) were obtained for the samples of each mixture after 28 days using 64 scans on average for each measurement at a resolution of 4 cm^−1^ in a range of 4000–400 cm^−1^.

## 3. Results and Discussion

### 3.1. Compressive Strength

**Figure 2:**
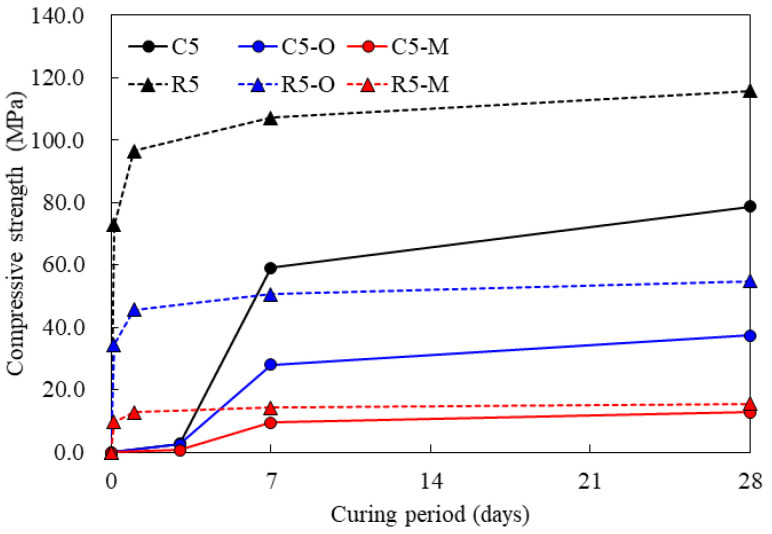
Compressive strength results.

Figure 2 shows the compressive strength of the various cement mortar mixtures prepared in this study. The mortar with OPC exhibited a significant increase in compressive strength in the middle and later ages. In addition, it was found that the compressive strength of the mortar with rapid-hardening cement increased rapidly in the early stages and continued to increase slightly in later ages.

With regard to the compressive strength, the rapid-hardening cement exhibited a higher strength than that of OPC. The compressive strength of OPC mortar at 28 days was found to be 78.7 MPa for C5, 37.4 MPa for C5-O, and 12.8 MPa for C5-M. Thus, the addition of water repellents to the mortar mixture with OPC reduced its compressive strength. Here, compared to the compressive strength of the mortar with no water repellent, the compressive strengths of mortar mixtures with oligomer and monomer water repellents were approximately 47% and 16%, respectively, indicating that the latter further reduced the strength of the mortar more.

The compressive strength of rapid-hardening cement mortar at 28 days was 115.8 MPa for R5, 54.8 MPa for R5-O, and 15.5 MPa for R5-M. The addition of water repellents to this cement mortar mixture type also reduced its compressive strength, as with OPC mortar. Here, compared to the compressive strength of the mortar with no water repellent, the compressive strengths of the mortars with oligomer and monomer water repellents were approximately 47% and 13%, respectively, indicating that the monomer water repellent reduced the strength of the mortar to a greater extent.

R5 and R5-O, which are samples of rapid-hardening cement mortar, developed more than 90% of the 28-day strengths of C5 and C5-O, which are samples of OPC mortar, at 3 h. In particular, R5-O developed approximately 70% of the 28-day strength of C5 despite the addition of the oligomer water repellent. R5-O also developed 92% of the 28-day strength of C5-O, in which the oligomer water repellent was added to the OPC mortar at 3 h, 122% at one day, and 146% at 28 days. The addition of the oligomer water repellent to the rapid-hardening cement mortar made it possible to rapidly develop and maintain a higher strength compared to that of the OPC mortar.

### 3.2. X-ray Diffraction (XRD)

**Figure 3:**
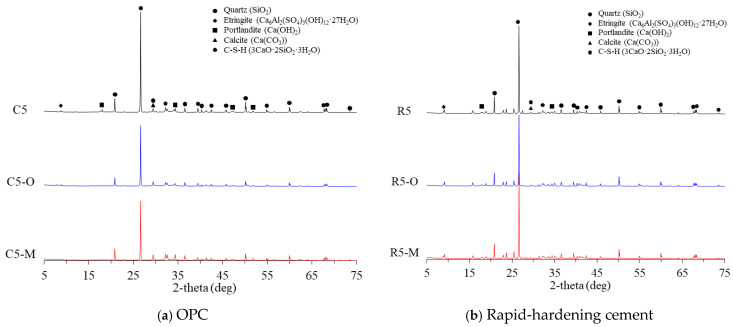
XRD results of cement mortar mixed with water repellents.

Figure 3 shows the XRD patterns of the cement mortar mixed with water repellent at 28 days of age. Typical hydration products, such as portlandite, ettringite, and quartz due to sand, were confirmed. As shown, the addition of water repellents resulted in no new reaction product, regardless of the cement type. A previous study also reported that cementitious materials mixed with silane/siloxane exhibited no difference in XRD from materials with no silane/siloxane after seven days [9].

As shown in Figure 3a, C5, the OPC mortar with no water repellent, shows a Ca(OH)_2_ peak at 2θ = 18°. It has been reported previously that portlandite improves the formation of C-S-H gel because it helps maintain the alkali pore solution concentration during cement hydration [16]. In the case of C5-O and C5-M with water repellents, however, the Ca(OH)_2_ peak was not observed, which appears to explain the development of lower compressive strength compared to that of C5.

In the case of the rapid-hardening cement mortar, all the mixtures of R5, R5-O, and R5-M exhibited the Ca(OH)_2_ peak. In addition, a peak corresponding to ettringite could be observed at 2θ = 9°.

### 3.3. Thermogravimetry-Differential Thermal Analysis (TG-DTA)

**Figure 4:**
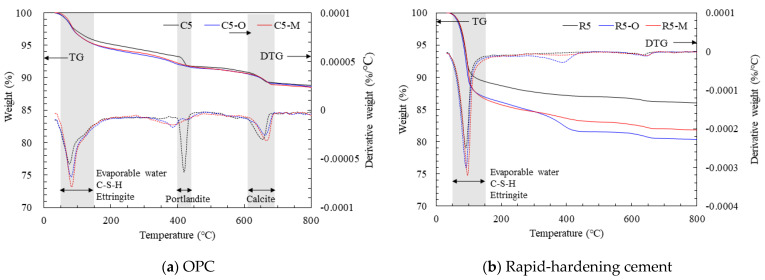
TG-DTG of the cement mortar mixtures with water repellents at 28 days of age.

Figure 4 shows TG-DTG results of the cement mortar with water repellents at 28 days of age. TGA curves indicate various phase transformations, such as C-S-H dehydration, formation of ettringite and AFm, and dehydroxylation of Ca(OH)_2_ [17]. Five main peaks in the mass loss regions of cementitious materials are observed according to the heating temperature: (1) evaporation of evaporable water and decomposition of C-S-H and ettringite, (2) decomposition of gibbsite, (3) decomposition of portlandite, (4) conversion of hematite, and (5) decomposition of calcite [18].

The main regions of the mass loss and differential scanning calorimetry (DSC) peak of hydrated compounds are 50–150 °C, 400–450 °C, and 650–800 °C. The decomposed compounds for each section are evaporable water, C-S-H, and ettringite in the 50–150 °C region; portlandite in the 400–450 °C region; and calcite in the 600–700 °C region. Portlandite was selected as an indicator of the hydration reaction because it is formed due to the hydration of C_3_S and C_2_S [19,20].

In the TG-DTG results of the OPC mortar in Figure 4a, a reduced mass was observed in the 50–150 °C region owing to the evaporation of water and decomposition of ettringite and C-S-H. Here, C5-O and C5-M exhibited a larger reduction in mass than that in C5, which appears to be caused by the unreacted moisture of the samples rather than by hydration products. In the 400–450 °C section, the decomposition of portlandite could be accurately determined by DTG curves for C5, and the mass loss caused by temperatures in the dehydroxylation reaction was clearly observed. C5 showed a mass loss of approximately 1.5% in the 400–450 °C region [16]. In contrast, C5-O and C5-M exhibited negligible mass losses of 0.5 and 0.6%, respectively. As with the XRD results, this result confirms that portlandite was hardly formed by adding water repellents to the OPC cement mortar. In the 600–700 °C region, all samples exhibited mass loss caused by calcite. Although the overall mass reduction was the same at the final heating temperature of 800 °C, the OPC mortar with no water repellent was affected by Ca(OH)_2_ reduction in the 400–450 °C section, and the mortar mixed with water repellents was affected by the reduction in evaporable water in the 100–150 °C region.

Figure 4b shows the TG-DTG results of the rapid-hardening cement mortar. All samples exhibited a mass loss in the 50–150 °C region as in the OPC mortar; however, the mass reduction was larger and the DTG curves were stronger. This appears to be due to the ettringite reduction observed in the XRD results of the rapid-hardening cement mortar. In the 50–150 °C region, the mass reduction rates of R5-O and R5-M were much higher than that of R5. This is due to the moisture loss, which was larger than the loss due to the hydration products, as the addition of water repellents caused the retention of a large amount of moisture, similar to the OPC mortar.

### 3.4. Water Contact Angle

**Figure 5:**
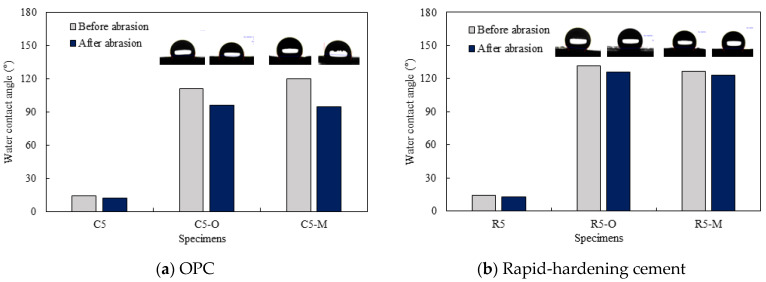
Water contact angle and water droplet shape of cement mortar mixed with water repellents.

Figure 5 shows the water contact angle and water droplet shape of cement mortar mixed with water repellents. Table 4 also shows the surface image of cement mortar at 160× magnification. As shown in Figure 5a, the contact angles were 111° for C5-O and 120° for C5-M; however, the contact angle could not be measured for C5 due to the absorption of water droplets on the surface. After surface abrasion, C5-O and C5-M exhibited contact angles of 96° and 94°, respectively, indicating that abrasion reduced the contact angle. As reported previously, siloxane and stearate concentrations in concrete are the highest near the surface [21]. Larger contact angles were observed on the surface without abrasion because siloxane or stearate moved to the surface along with water during drying.

As shown in Figure 5b, the contact angles were measured to be 132° for R5-O and 126° for R5-M; however, the contact angle could not be measured for R5, as the water droplets were absorbed on the surface. After surface abrasion, the contact angle decreased to 126° for R5-O and 123° for R5-M, but the reduction rates were less than 5%. As can be seen in Table 4, the OPC mortar and rapid hardening cement mortar with the water repellent showed a rough surface with the sand exposed in the abrasion; however, the contact angle of the super-velocity cement mortar was still greater than 120°. The rapid-hardening cement mortar mixed with water repellents exhibited a larger water contact angle than that of the OPC mortar. The former improved the internal hydrophilicity while maintaining the contact angle even after abrasion.

### 3.5. Water Absorption Coefficient

**Figure 6:**
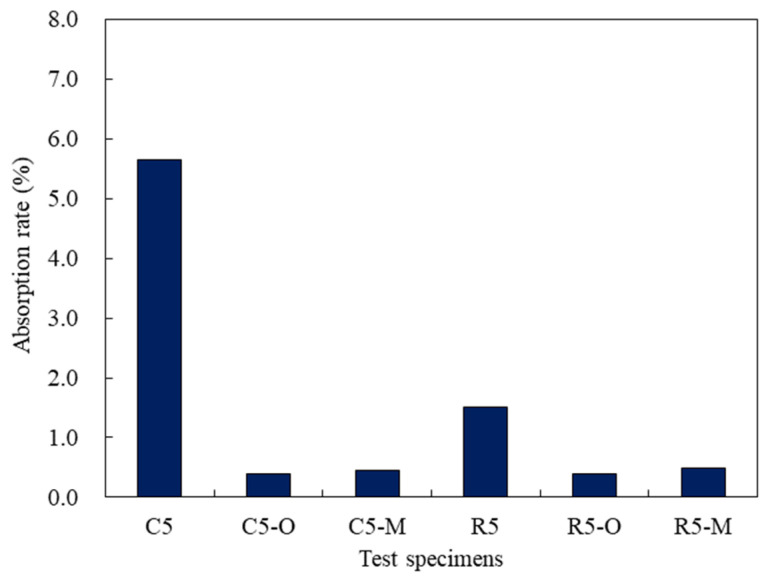
Water absorption of the cement mortar mixed with water repellents.

Figure 6 shows the water absorption of the cement mortar mixed with water repellents. The water absorption rates were found to be 5.65% for C5, an OPC mortar with no water repellent, and 1.51% for R5, a rapid-hardening cement mortar. It was less than 1% for the cement mortar mixed with water repellents for all cement types. The water absorption of the cement mortar was significantly less because water repellents prevent water penetration by forming a smooth film on the mortar samples [22]. The water repellents developed hydrophobicity in the cement mortar and effectively prevented the infiltration of external water. They maintained a very low absorption rate even though the strength of the cement mortar was reduced. Mortar without resin has a very low water-carrying capacity, but resin-like mortar exhibits a high hydrophobicity. Organosiloxane changes the hydrophilic surface of the capillary to a hydrophobic surface because it contains several hydrocarbons in its structure [14]. This appeared to improve the hydrophobicity of the surface without water absorption.

The capillary water absorption of the mixed mortar may vary depending on the distribution inside the mortar matrix, as well as the mixture composition. The cement mortar mixed with water repellents did not absorb water due to hydrophobicity on the surface and in the body, which conflicts with the general tendency that the water absorption rate increases as the strength decreases. The finding that the water absorption rate was not proportional to the strength indicates that water repellents were well bonded inside the mortar. Cement mortar mixed with water repellents is expected to contribute to the mortar durability, such as freezing and thawing, owing to the very low water absorption rate.

**Figure 7:**
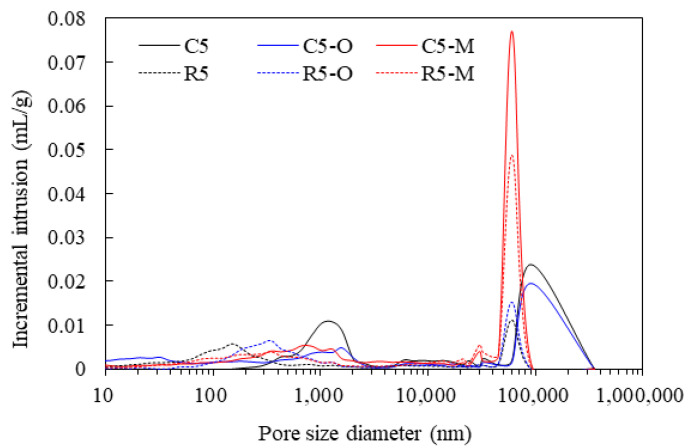
Pore size distribution of the cement mortar mixed with water repellents.

The pore structure related to water absorption capacity is shown in Figure 7. In the figure, in particular, in the case of the cement paste with the addition of the monomer water repellent, it was confirmed that pores as large as 10,000 to 100,000 nm were created, but the cement paste with the monomer water repellent showed less than 1% absorption. In the results of the absorption rate measured in a short period of time, the effect of the water repellent was greater than the effect of the pores. In addition, the pore size diameter result can prove that the cement mortar added with the monomer water repellent shows very low strength in the compressive strength result shown in Figure 2.

### 3.6. Fourier-Transform Infrared Spectroscopy (FT-IR)

**Figure 8:**
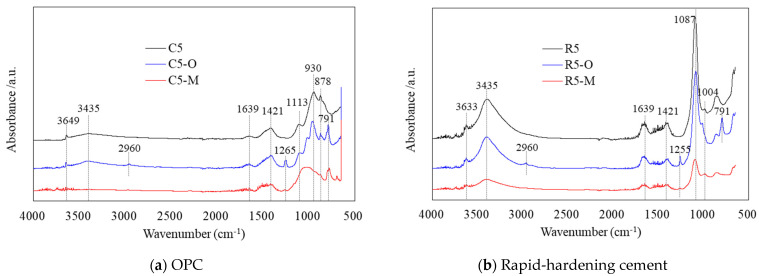
FT-IR spectra of the cement mortar mixed with water repellents.

Figure 8 shows the FT-IR spectra of the cement mortar mixed with water repellents. FT-IR characterization was used to verify the chemical bonding of the added water repellents. Due to the absorption of long aliphatic chains of stearate, a barrier to normal hydration can be constructed owing to the formation of a water-repellent layer on the clinker surface [23,24].

In Figure 8a, the FT-IR peaks of the hydrated OPC mortar show O–H stretching at 3435 and 3649 cm^−1^, O–H bending at 1639 cm^−1^, C–O stretching at 1421 and 878 cm^−1^, S–O stretching at 1121 cm^−1^, and Si–O stretching at 930 cm^−1^. The peaks of Si–O–Si due to the aggregate of mortar could be observed at 1105 and 950 cm^−1^, but it was difficult to confirm that they overlapped with the peak of Si–O stretching.

In the case of C5-O, in which the oligomer water repellent was added to the OPC mortar, the presence of siloxane –Si-CH_3_ was confirmed because a –CH_3_ stretching peak at 2975 cm^−1^ and Si–CH_3_ stretching peaks at 1265 and 791 cm^−1^ were observed. For C5-M, in which a monomer water repellent was added to the OPC mortar, –Si-CH_3_ could be confirmed as Si–CH_3_ stretching peaks were observed near 1265 and 800 cm^−1^.

In particular, C5-O with a water repellent exhibited low O–H stretching peaks at 3435 and 3649 cm^−1^ compared to those of C5 with no water repellent. In the case of C5-M, however, O–H stretching peaks could hardly be observed, indicating quite low amounts of hydration products. This result is consistent with the very low amount of portlandite observed in the XRD and TG-DTG results.

In Figure 8b, the hydrated rapid-hardening cement mortar shows a very high peak strength, unlike that of the OPC mortar. The FT-IR peaks of the hydrated rapid-hardening cement mortar showed O–H stretching at 3435 and 3633 cm^−1^, O–H bending at 1639 cm^−1^, C–O stretching at 1430 (1421) cm^−1^, S–O stretching at 1113 (1087) cm^−1^, and Si–O stretching at 1004 cm^−1^. As with OPC mortar, the peaks of Si–O–Si caused by the aggregate could be observed at 1105 and 950 cm^−1^, but the overlap with the peak of Si–O stretching was difficult to confirm.

In the case of R5-O, in which the oligomer water repellent was added to the rapid-hardening cement mortar, –Si-CH_3_ of siloxane could be observed as –CH_3_ stretching at 2960 cm^−1^ and Si-CH_3_ stretching peaks at 1255 and 791 cm^−1^ were observed. For R5-M with the monomer water repellent, no Si-CH_3_ stretching peak was observed.

For R5-O and R5-M, in which water repellents were added to the rapid-hardening cement mortar, the O–H stretching peak was observed at 3435 cm^−1^. In particular, R5-O clearly shows an O–H stretching peak at 3633 cm^−1^. As confirmed by the XRD and TG-DTG results of the rapid-hardening cement mortar, the O–H stretching peaks appear to consist of hydration products including ettringite. The large amount of hydration products, as confirmed by the high and wide peaks, indicates a compressive strength higher than that of the OPC mortar with water repellents.

## 4. Conclusions

In this study, we attempted to fabricate cement mortar mixtures that enable quick emergency repair and maintain hydrophobicity, regardless of time and abrasion, by mixing the mortar with water repellents. Upon doing so, hydrophobicity was found to develop on the surface and inside the samples despite a reduction in compressive strength. It is expected that this method of adding a water repellent when manufacturing the mortar can better improve a structure’s resistance to moisture penetration compared to the surface-coating method. In this study, the mortar mixtures prepared by adding oligomer water repellent to rapid-hardening cement exhibited excellent performance in terms of strength and hydrophobicity. Therefore, these mixtures are suitable for application as a repair material in watertight structures that require rapid construction. In the future, we will investigate the pore structure and water absorption rate of cement mortar due to the addition of silane and siloxane as well as the economic efficiency of the rapid-hardening cement mortar with hydrophobicity.

## Figures and Tables

**Table 1:** Physical properties and chemical composition of ordinary Portland cement (OPC).

Type	Blaine (cm^2^/g)	Setting Time		Density (g/cm^3^)	Chemical Composition (%)						
		Initial (min)	Final (hour)		SiO_2_	Al_2_O_3_	Fe_2_O_3_	CaO	MgO	SO_3_	lg. Loss
Ordinary portland cement	3300	200	5.5	3.15	21.7	5.7	3.2	63.1	2.8	2.2	2.4
Rapid-hardening cement	5741	25	0.67	2.91	13	19	3	48	2.5	11	2

**Table 2:** Physical properties and chemical composition of water repellent.

Type	Active Ingredients (wt%)	Density (g/cm^3^)	pH
Oligomer	60 or more	1.01	6–8
Monomer	40 or more	1.01	6–8

**Table 3:** Experimental plan.

Type of Binder	Water Repellent	Mixed Design	Test Parameters
Ordinary portland cement (C) Rapid-hardening cement (R)	Oligomer (O) Monomer (M)	B:S:W:R = 40:40:15:5	Compressive strength X-Ray Diffraction TG-DTG Water contact angle (before and after abrasion) Water absorption FT-IR

**Table 4:** Surface images.

	C		R	
	Before Abrasion	After Abrasion	Before Abrasion	After Abrasion
Additive-free	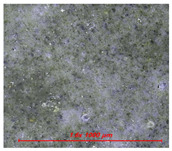	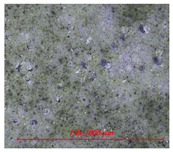	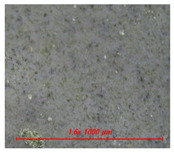	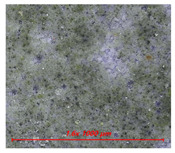
O	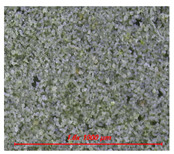	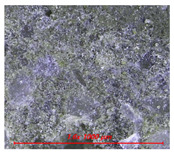	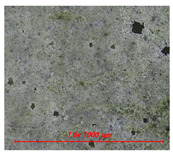	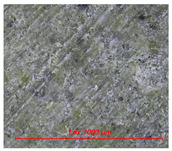
M	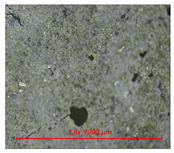	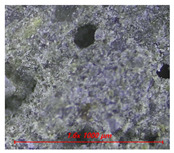	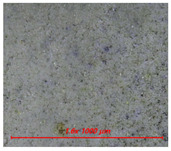	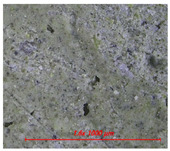

## Data Availability

The data presented in this study are available on request from the corresponding author.
